# Adaptive strategies used by surgical teams under pressure: an interview study among senior healthcare professionals in four major hospitals in the United Kingdom

**DOI:** 10.1186/s13037-024-00390-3

**Published:** 2024-02-21

**Authors:** Dulcie Irving, Bethan Page, Jane Carthey, Helen Higham, Shabnam Undre, Charles Vincent

**Affiliations:** 1 Department of Experimental Psychology, University of Oxford, Oxford, UK; 2 Cicely Saunders Institute, King’s College London, London, UK; 3 Jane Carthey Consulting, Oxford, UK; 4 Nuffield Department of Anaesthetics, Oxford University Hospitals NHS Foundation Trust, Oxford, UK; 5 Department of Urology, East and North Hertfordshire NHS Foundation Trust, Stevenage, UK

**Keywords:** Health services, Healthcare, Surgery, Teams, Pressure, Crisis management, Adaptation, Strategies

## Abstract

**Background:**

Healthcare systems are operating under substantial pressures, and often simply cannot provide the standard of care they aspire to within the available resources. Organisations, managers, and individual clinicians make constant adaptations in response to these pressures, which are typically improvised, highly variable and not coordinated across clinical teams. The purpose of this study was to identify and describe the types of everyday pressures experienced by surgical teams and the adaptive strategies they use to respond to these pressures.

**Methods:**

We conducted interviews with 20 senior multidisciplinary healthcare professionals from surgical teams in four major hospitals in the United Kingdom. The interviews explored the types of everyday pressures staff were experiencing, the strategies they use to adapt, and how these strategies might be taught to others.

**Results:**

The primary pressures described by senior clinicians in surgery were increased numbers and complexity of patients alongside shortages in staff, theatre space and post-surgical beds. These pressures led to more difficult working conditions (e.g. high workloads) and problems with system functioning such as patient flow and cancellation of lists. Strategies for responding to these pressures were categorised into increasing or flexing resources, controlling and prioritising patient demand and strategies for managing the workload (scheduling for efficiency, communication and coordination, leadership, and teamwork strategies).

**Conclusions:**

Teams are deploying a range of strategies and making adaptations to the way care is delivered. These findings could be used as the basis for training programmes for surgical teams to develop coordinated strategies for adapting under pressure and to assess the impact of different combinations of strategies on patient safety and surgical outcomes.

**Supplementary Information:**

The online version contains supplementary material available at 10.1186/s13037-024-00390-3.

## Background

The pressures on health services in the United Kingdom (UK), United States and other countries around the world are increasing, in part exacerbated by the aftermath of the Covid-19 pandemic [1–4]. As a result of these pressures on health systems, the quality of care may fall short of the standard expected by patients and aspired to by healthcare professionals [5, 6].

Surgical teams adapt to these pressures, and in the great majority of cases, achieve good outcomes despite adverse circumstances. However, when demand exceeds capacity, risk to patients and the burden on staff inevitably increase. Surgical teams adapt in a variety of ways, but the adaptations are usually improvised and vary widely depending on who is in charge at the time [7]. We have previously argued that we need to define a portfolio of strategies that clinical leaders can deploy when pressures are high, and that an explicit and coordinated approach to adaptations will be safer than uncoordinated improvisation [7].

To explore the nature of adaptations to care at times of pressure, we recently conducted a review of the resilient healthcare studies which used interviews and observations of teams under pressure. The data described in these studies was used to develop a taxonomy of pressures and strategies used to respond to these pressures [8]. Teams and individuals adapted care in many different ways, using strategies for adapting on the day and anticipatory strategies to plan for future pressures. Adaptations included different forms of prioritization of care, changes to working practices and usual procedures such as adjusting staff-patient ratios or creating temporary holding spaces for patients. This review however contained very few studies specifically describing surgical environments [5, 6].

The aim of the current paper is to explore the types of everyday pressures experienced in surgery in UK hospitals, and to identify the ways in which surgical teams adapt clinical practice to meet demand while managing the risk to patients. This study focuses on the specifics of what measures staff working in surgery take to adapt when under pressure, with the longer term aim of defining a portfolio of adaptive strategies that clinical leaders might employ and evaluate in their own hospitals.

## Methods

### Study design

Semi-structured interviews were conducted with senior healthcare professionals working in four hospitals across England to identify the types of everyday pressures experienced by individuals and teams working in surgery, and the adaptive strategies they use to respond to these pressures. This project was reviewed by the Oxford University Hospitals NHS Foundation Trust Joint Research Office and classed as a service evaluation. As such, it was not subject to the Department of Health’s UK Policy Framework for Health and Social Care Research (2017) and a full ethics review.

### Sampling and setting

Four acute hospitals from across England were purposively sampled to capture different geographical locations, size and type of hospital and population demographics. Two were large Teaching hospitals and two were District General Hospitals. Data collection was completed between September 2022 and August 2023.

We identified a lead contact known to the researchers in each hospital for consultation, suggestions of interviewees and communication of findings. We approached 29 members of surgical teams, with 20 agreeing to be interviewed and no response from nine. We interviewed 20 participants with a sample that consisted of Surgeons (*n* = 4), Anaesthetists/Anaesthesiologists (*n* = 6), Matrons/Clinical Nurse Managers (*n* = 4), Senior Registered Nurses (*n* = 3) and Theatre Managers or similar (*n* = 3). Participants had a mixture of experience working in elective and emergency surgery covering a broad range of specialties, including head and neck surgery, trauma surgery, maxillofacial surgery, paediatric urology, breast cancer or breast reconstruction, colorectal surgery, general children’s surgery and general surgery.

### Data collection

A draft interview guide was developed with the aim of collating specific examples of strategies that individuals, teams and organisations use to cope with everyday pressures (Additional file 1). The interview questions were informed by the findings from our recent scoping review on resilient healthcare conducted by our team [7], and adapted in response to two pilot interviews. Participants were invited to take part via email and sent a full information sheet. Verbal consent was obtained at the beginning of each interview, which included permission to record the interviews for the purpose of generating a transcript. Interviews were conducted by a human factors and patient safety consultant (JC) and two researchers experienced in qualitative methods and healthcare research (BP and DI). Semi-structured interviews were conducted over video call, audio-recorded and transcribed verbatim. Field notes were taken during the interviews to follow-up on points of interest or seek clarification. Each interview lasted approximately 1 hour.

### Analysis

The data was analysed using a thematic template approach [9]. The qualitative data management tool NVivo was used to manage and code the interview data. The first stage of analysis was data familiarisation: BP and DI read the interview transcripts and shared initial reflections and preliminary coding strategies during a series of meetings with other members of the research team (JC & CV). The second phase was to create and iteratively develop a framework for organising the data, drawing on the interview guide and the taxonomies of pressures and strategies developed from the scoping review conducted by our team [8]. These taxonomies provided a framework for organising the pressures and strategies described in the interviews. The coding framework was piloted on a sample of four interviews and was found to capture the key pressures and strategies described. Data from each transcript were then indexed through systematically coding quotations and placing them in one (or more) of the framework categories. A small number of categories from the framework were not present in the data as they were not relevant to surgical care. Completion of these steps provided a manageable data set to analyse. BP and DI led the data analysis, and JC and CV provided oversight. The research team met regularly to discuss the analysis and coding framework.

## Results

### Pressures

**Fig. 1:**
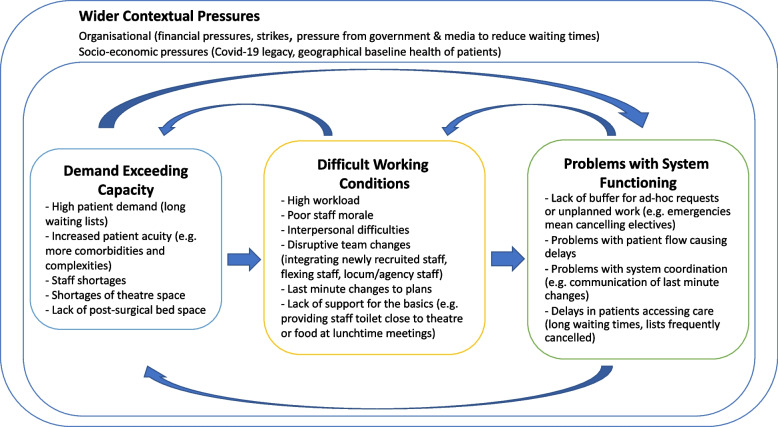
Interrelating pressures commonly experienced in surgery, adapted from Page et al. [8]

The pressures reported by clinical staff in the interviews could be broadly categorised into (i) demand exceeding capacity; (ii) difficult working conditions and; (iii) problems with system functioning. High demand makes working conditions difficult which in turn places more pressure on the whole system of care. Although we categorised pressures into three separate groups, there are, in reality, multiple feedback loops and interactions between the different pressures and problems. For example, delays at various points in the patient journey, make it much more difficult to cope with new patients coming into the system. Figure 1 demonstrates this dynamic process and summarises the principal pressures described by surgical teams.

### An overview of adaptive strategies

In the face of these pressures, there was commonly a tension between safety and efficiency, and participants described a pragmatism about the decisions and choices they had to make on a daily basis. In this section, we describe the strategies staff used to adapt in response to pressures, both to prepare in advance (anticipatory strategies) and to adapt on the day to manage immediate pressures, although the line between these two was not always clear-cut in surgery. Strategies employed can be divided into three broad categories: surgical teams may increase or flex resources, they may control or prioritise demand or they may make adjustments to service delivery by optimising efficiency and adapting communication, leadership and teamwork. In the following sections we describe the many forms of adaptation, both anticipatory and on-the-day, within these three broad categories and provide some illustrative quotations from interviewees.

### Increasing or flexing resources

Participants described some innovative strategies for addressing shortages of staffing and of skills (Table 1). Strategies included efforts to increase the level of competency of incoming staff and upskilling existing staff to be able to take on additional responsibilities. One hospital experienced a recurring on-the-day pressure of needing to send scrub nurses to sterile services to help with issues processing instruments. In response, they brought the two teams together to develop a shared understanding of the processes to improve skills and increase efficiency rather than waiting for the problem to occur on the day (Table 2). Day-to-day, teams relied on staff taking on extra shifts or staying late to cover staff sickness and increased patient demand. Scrub nurses and anaesthetists were often moved around between theatres to cope with shortages; however, because some types of surgery are highly specialised (e.g. maxillofacial surgery) there were limits to how flexibly teams can deploy staff.

*“So, they [student nurses] are coming to us on a final placement, which means they are in third year, beginning April. Then they spend three months with us. So, what we did is we modified their competency, so it matches the requirement, what's the minimum set that the university requires, and matched them with our Band 5 [newly qualified nurse] competency. So, effectively, they're like on-the-job training. So, those nurses or trainee nurses on their final placement in April, but the time they qualify in September and ready to start with us, if we manage to convince them, then that means their supernumerary period would be a lot shorter. So, in a way, we try to speed up the process.*” [Matron]

**Table 1:** Examples of strategies used by surgical teams to increase and flex resources

Theme	Strategy	Example
**Adaptations to staffing and skill mix**		
Anticipatory	Increasing numbers of incoming staff that are already skilled	Work closely with the local university to develop and adapt teaching courses (e.g. additional bespoke module in anaesthetics for scrub/recovery nurses) Increase final year placements to improve skills and experience of nursing students, thereby reducing supernumerary period for new starters
	Diversifying job roles to shift the workload away from the consultants	Recruiting and training more physician associates to run their own lists (overseen by consultant anaesthetist). Maximise efficiency by allowing anaesthetist to support activity in more than one theatre Specialist nursing associates and junior doctors trained to independently run clinics, pre-assessments and initial consultations with patients, freeing up surgeons or anaesthetists to spend more time in theatre
	Improving skill mix in theatre through staff rota planning	Requirement that senior nursing staff are available to supervise and teach new staff assisting in surgery. Rotational plan of newly qualified and international recruits in surgery to ensure adequate support can be given by senior nurses
	Pooling patients (operated on by any surgeon or any surgeon within a specialty)	The cases under a specific surgeon who are approaching the maximum wait time are assessed to see if they can be pooled, and if a different surgeon is willing to take the case
On-the-day	Task-shifting or extension of responsibilities to share workload	Junior doctors starting a list while the consultant is busy with a patient or resolving scheduling issues Communicating with families delegated to who is available so that senior surgeon and anaesthetist can remain in theatre Nursing staff taking on the role of junior doctors, or healthcare assistants taking on the role of a scrub nurse
	Flexing staff to address numbers or skill mix issues	Scrub nurses sent to help sterile services process instruments quickly to prevent delays and disruption Senior nurses in non-clinical roles provide patient care to cover sickness and absences Staff asked to help with an emergency case if their elective list has finished early
**Adaptations of space, beds, services and equipment**		
Anticipatory	Providing more services to reduce high patient numbers by organising extra clinics or surgical lists	Adding extra patients into clinics (reducing appointment time/accept overrunning) or organising additional clinics and surgical lists to help with the backlog (e.g. on a weekend). This often involved staff working overtime and was contingent on space and all the necessary staff being available to run it, which was not easy to organise
	Reorganising systems for the processing of instruments in advance to maximise efficiency on the day	Scrub nurses redeployed to the sterile services team to understand the process of packing instruments and to educate the sterile services team on which instruments are opened together for specific cases. Sterile services staff observe in theatre so they have context when prepping instruments for each surgical procedure. The aim being to have a better shared understanding of how to pack instruments in a way that fits with the flow of the surgical procedure and prevent delays and disruption on the day. Anything else required must be requested by the surgeon in advance
On-the-day	Flexing space by repurposing theatres for different types of surgery, described as “flipping theatres” or “ad hoc theatres”	Theatres being used for different types of surgery to the norm, providing the space and equipment was suitable. For instance, theatres for a list stood down due to staff unavailability would be given to a different specialty
	Providing care in non-standard areas when beds are unavailable on the ward	Due to a lack of bed space on the wards, patients might be kept in the recovery room for longer and sometimes cared for by the staff there overnight Staff might admit male patients to the female ward (and vice versa) when there were a lack of beds

**Table 2:** Case study: process improvement in response to pressures

*“But I think the biggest problem is sterile services, is how they process the instruments. Sometimes, there was few times that we as a scrub team, we have to arrange a staff to go and help them to process instruments just not to cancel any cases or procedures or prioritised, especially the cancer patients. So I think that happened because of their lack of skill mix as well and at the same time sicknesses on the day…*
*So we've arranged orthopaedic skilled staff to help them out, for example, like in the morning, they can go and process some of the basic instruments, and then in the afternoon, they could come back to theatres to help out as well. So at least just to process the basic stuff for us to carry on…*
*So we found out that it was really helpful on their part, because they could see, for example, not to compare their staff and our staff like, for example, their orthopaedic staff could manage to pack or process ten instruments within an hour. For example, and CSSD [Central Sterile Services Department] staff, they could only process two in one hour. Yes, so that makes a big difference on their part. Yes, I think we discussed that with the managers and the team as well.”* [Lead Theatre Practitioner]

Theatre space was a key constraint. Sometimes theatres were used for different types of surgery to the norm to meet the needs of the service (e.g. emergency surgery performed in an elective theatre that had finished early). Again, this was not always straightforward and depended on the skillset of staff and availability of equipment needed in that theatre. Other strategies to increase capacity for the level of demand included arranging extra clinics or surgical lists, and caring for patients in recovery rooms or the post-anaesthesia care unit (PACU) for longer when beds were short on the ward (Table 3).

**Table 3:** Case study: intervening with short-term gains but potential unforeseen negative side effects

*“Some of the strategies we have from elective perspective, if we are looking to be very short from an elective bed perspective, it's like, can recovery staff the recovery suite overnight? It's not great from a patient experience perspective because recovery is designed as recovery, there's no functioning bathrooms or anything down there. But to balance that, it prevents us cancelling an operation. And the patients are usually very grateful to have spent a night in recovery because they've got their operation and not had to be sent home…*
*They know there's a bed crisis, because patients in surgical reception are very aware of the communication and everything that's going on. So some patients are just so grateful that they have had their operation. Other patients aren't, and they get frustrated by the lack of facilities, mainly, the access to food, although, we have greatly improved that, we do try to make sure that our patients get well fed if they are outside of a ward area…*
*But when you are co-locating patients who are going to spend overnight in recovery next to patients who are just coming out of theatre, post anaesthetic, they’re very different environments. You've got a patient that's recovered, and you've got a patient who's just about to start their recovery journey. And they're very different environments…If you're a patient that's been there for four hours, you're completely aware of what's going on around you, visually, you see patients coming through with tubes in their mouth, and drips and drains, and it's not a very nice experience…*
*And sometimes for the staff in recovery, it's actually quite difficult, because they are used to looking after patients who are asleep, essentially, who will then become drowsy and awake and will go to a ward…And that's a real challenge then for the staff to be able to manage. So there's lots of downsides, but there's lots of upsides, for want of a better word, as well, because patients have been able to have their procedure.”* [Ward Matron]

### Controlling and prioritising demand

The principal means of controlling patient demand was to either cancel surgery or to schedule fewer operations, although the manner in which this was done varied. If surgery needed to be cancelled, staff aimed to inform patients in advance, but sometimes unforeseen issues (e.g. emergencies, staff sickness or patient factors) meant surgery was cancelled on the day of the operation. Surgical teams might delay cancellation in the hope of finding another solution, such as drawing staff from other areas.

*“I’m pretty sure one list is going to go down because there’s no anaesthetist. So, that cut us one. And the other one, it’s next Friday. It’s another world. I’m going to leave that decision until Wednesday. Now, I’ve clocked it for Wednesday when we have our theatre planning meetings. But do you know what? I think we’ll probably get that covered by then. And if I were to react today and say, right let’s stand down the list, the chances are we would lose a list and patients would be cancelled. We would get an anaesthetic practitioner by Thursday and that list could have gone ahead. So, it’s often a balancing act by not reacting to something. It’s dangerous.”* [Theatre Manager]

These decisions were stressful for teams who are balancing the patient’s need for surgery with the risk of letting them down at the last minute. Patients are prioritised based primarily on clinical need (e.g. cancer and emergency patients), as well as waiting time, but individual circumstances were also considered (e.g. impact on daily life, distanced travelled). These criteria are applied to prioritising patients in advance as well as on the day if theatre space became available. Decisions also had to be made based on whether delaying the surgery by a few days might affect the patient’s mortality or longer-term outcomes. A range of reported strategies used to control and prioritise demand is shown in Table 4.

**Table 4:** Examples of strategies used by surgical teams to control and prioritise demand

Theme	Strategy	Example
**Control Demand**		
Anticipatory	Suspending or restricting services through planned cancellations of surgery	Cancelling elective surgery or standing down theatres used for less urgent surgery to make way for emergency surgery or fast-track patients Doing fewer surgeries in anticipation of forthcoming pressures in a few days’ time
	Discharging patients sooner from surgical wards to create space for anticipated need	If the surgical ward is full, patients for anticipated discharge tomorrow are reviewed today to expedite discharge to make beds available for emergency patients the next day
On-the-day	Cancelling surgeries on the day	Incoming emergencies or staff sickness meant surgeries were cancelled on the day
	Restricting admissions to the ward	Restricting additional patients coming onto a full ward by having them cared for in recovery for longer, and start times for the next surgeries are pushed back
**Prioritising Demand**		
Anticipatory	Referring to national systems for prioritising patients by clinical need and wait time	Patients are prioritised from P1 (most urgent) to P4 (least urgent). P2 would include cancers and urgent surgery; P3 patents should be seen within 3 months. Patients who had had their surgery cancelled should be rescheduled for surgery within 28 days
On-the-day	Prioritisation of patients when theatre space became available on the day	Ad hoc meetings of surgeons or multidisciplinary team discussions, to decide which patients ought to be prioritised when a spare theatre or post-operative bed became available on the day

### Strategies for managing the workload

Participants described a range of strategies for adapting their usual ways of working to manage high levels of pressure and demand (Table 5). Adjustments to scheduling of theatre lists was a widely used strategy. Lists were carefully planned in advance, ideally collaboratively between surgeons, anaesthetists and theatre managers (and schedulers), to optimise theatre time and minimise last minute changes or cancellations. At times new teams will be created at very short notice to manage rapid changes and demand, requiring coordination across the multiple different disciplines involved (Table 6). Surgeons and anaesthetists described how they would often look to see which members of staff had been allocated to the list, which enabled them to have a realistic expectation of their day and be able to plan and adapt for it accordingly.

*“So, when I look at my list and I say, this list is too long. And they say, oh, but we need to fully occupy the theatre. I said, who is my anaesthetist? So, if I know who my anaesthetist is in advance then I can accept the few extra minutes of operating because I know between us we will make up the time. But if I don’t know who the anaesthetist is or I have a very slow one then there’s no way it’s going to happen.*” [Surgeon]

**Table 5:** Examples of strategies used by surgical teams to manage the increased workload

Theme	Strategy	Example
**Scheduling for efficiency**		
Anticipatory	Keeping elective and emergency theatres and pathways separate	Keeping elective and emergency theatres and pathways separate protected the scheduling of elective surgery and prevented delays in emergency surgery by having separate bed bases for each set of patients
	Having a ‘six-four-two weeks out’ rule for creating operating lists and allocating staff	The ‘six-four-two’ rule where surgeons and anaesthetists are assigned six weeks out, patient lists created four weeks out, staff and lists finalised two weeks out. If this cannot be fulfilled by two weeks, the theatre is offered to another service and anaesthetist is reassigned
	Theatre managers, anaesthetists, surgeons and other members of the multidisciplinary team working collaboratively to plan lists	Planning of lists was done collaboratively with surgeons, anaesthetists and other members of the multidisciplinary team to maximise efficiency, minimise last minute changes and have an accurate estimation of timings to avoid overrunning or cancellations
	Stand-by patients who are pre-assessed and ready to come in if there are cancellations	Increase pre-operative capacity to have a pool of standby patients ready to come in (e.g. not had breakfast) to reduce wasting theatre time if a surgery is cancelled
	Looking at staff rosters in advance to anticipate necessary adjustments and allowances	Surgeons and anaesthetists look at staff allocation to the list ahead of time to anticipate pressures related to timing and level of supervision. For example, having an experienced trainee in theatre would determine operating time or allowances for anaesthetist to take unexpected requests such as assessing emergency patients
On-the-day	Renewed emphasis on strict start times for all theatre staff	All the operating team to meet promptly at the agreed start time to reduce late starts, late finishes and staff being idle waiting for others to arrive, enabling the day to flow as planned
	Filling in short gaps in lists with simple standby local anaesthetic (LA) or emergency cases	Space that has become available during the day may be filled with simple cases to optimise theatre time and staff that are available in normal working hours. A “quick LA” may also be arranged at the start of a list while general anaesthetic case reviews are taking place before the team brief
**Communication and Coordination**		
Anticipatory	Planned multidisciplinary team meetings to coordinate plans between disciplines or teams from different sites	Having an up-to-date knowledge of resources and demand was critical for optimising the allocation of staff and scheduling for theatres. Planned multidisciplinary team meetings were vital for these updates when it might not be easy to communicate ad hoc on the day itself when individuals are elsewhere
	More attention to detail in email chains and making sure all relevant people are copied in	Theatre managers emphasised the importance of attention to detail in email communication about changes to lists or staffing, to ensure all the right people were copied in. This prevented pressures of errors and confusion on the day. For example, the staff rota has changed but relevant staff haven’t been informed
	Planning how and when to communicate plans with patients	Sharing with patients a realistic or slightly longer than anticipated waiting time, to prevent disappointment and additional workload for staff receiving calls from upset or chasing patients Implementation of a new policy to only inform patients of their scheduled date for surgery two weeks before to minimise the numbers of cancellations and changes
On-the-day	Adaptations in communication style when pressures increase	Speaking slower and softer when under pressure in theatre to focus the attention of others More use of closed-loop communication to check understanding in order to prevent errors or time delays
	Multimodal communication and increase in face-to-face contact	Although difficult when staff are in different theatres, participants emphasised importance of face-to-face communication when pressures are high to harness support and request help where needed
	Using other means of quick communication	Use of WhatsApp for communicating changes and problems on the day to maintain streams of communication
**Leadership**		
Anticipatory	Increased emphasis on creating an environment of psychological safety	Being approachable and being explicit that people should feel able to speak up and communicate any concerns or questions. This was thought to improve performance, patient safety, staff wellbeing and leaders having good oversight of the situation
	Reinforcing professional autonomy within the team	Reinforcing professional autonomy so everyone felt equally valued. For example, one surgeon had a rule that doctors are not to take from the nurses’ trolley or store supplies there without asking them first. Having boundaries like this helped to prevent pressures arising from interpersonal difficulties within the operating theatre team
	Adapting pace of work to the skills and expertise of the team	Knowing the skills and expertise of the team enabled leaders to be able to delegate, allocate roles suitably and take precautions. For example, notifying a surgeon of a new team member so they can plan to go more slowly or take special precautions
	Staff support initiatives	Supporting staff by allocating mentors and organising team lunches to help build psychological safety
On-the-day	Being more directive and autocratic when communicating decisions and being willing to be unpopular	One anaesthetist explained that when they make a decision on the day about which emergency patient will get the theatre, they make it as near final a decision as possible and did not change their mind easily to limit errors and distractions based on last minute alterations
	Pausing operations to regain situational awareness	Stepping back from the situation and looking at the whole system rather than the person shouting the loudest. One surgeon described physically leaving theatre to take a breath, regroup and review with a fellow surgeon. Interviewees also described the importance of focusing on the patient or situation in front of them and not getting distracted by potential problems down the line that were beyond their influence
	Staff member allocated as leader/coordinator of the day	‘Of the day’ role for who was in charge of operations and resource allocation for that day (e.g. matron of the day, operational senior nurse of the day, ‘silver bleep holder’). This meant there was a designated person with decision-making authority whose role it is to troubleshoot everyday pressures
	Providing support to staff on the day	Making sure the team were taking their breaks and foster an environment where staff feel able to question or ask for help. Clinical leaders would also organise additional debriefs or provide positive feedback at the end of a difficult day. This was to resolve additional stress and help prevent burnout
**Teamwork**		
On the day	Periodically regrouping and reviewing the plan and reallocating roles to manage changing pressures	Setting aside the theatre team hierarchy and empowering staff with the relevant skills and expertise to lead problem resolution in response to changing conditions and emerging pressures. For example, a healthcare assistant guiding an education team nurse covering staff absence on setting up the equipment needed in a particular theatre when surgical plans changed for the patient on the day
	Shared decision making and asking for help from others	Interviewees often expressed the importance of using the team and not feeling alone. Both nursing and medical staff emphasised the need to redirect or delegate to share out the workload when their own capacity was reached. Asking for help from others and sense checking to review difficult decisions were also key strategies

**Table 6:** Case study: improvised teamwork to create temporary capacity

*“We had already got three hip fractures admitted from the night before, so they were on our list, and we had another four hip fractures come in, in the same afternoon. So, on the day those three were being done, another four came in. So I happened to be on call, so I sent a message in the WhatsApp group saying I’ve got three hip fractures and a fourth coming into the ED department*
*Our orthogeriatric team picked up on that, and they immediately came to the Emergency Department to see the patients. My trauma registrar colleagues picked up on it, and a couple of them came and did the consenting while I did the history-taking and examinations. So, making sure everything’s okay while they just did the consent and booked the patient into theatre, because they were going to be operating anyway. And they called the anaesthetist to come and see. So that worked really well, everyone just pulled their weight and got on*
*Because we knew we were going to have a busy week, I looked at the theatres list for the next day, knowing that there’s no way we could get all those cases done that day. We found a list that had gone down, so I spoke to theatre staff and asked them if they could maintain that list that had been brought down, if they had enough staff. Even though that list had dropped two weeks before, we weren’t notified about it*
*But they had enough staff to run a skeleton crew to keep that theatre running the next morning, so I stayed behind the next morning to finish the list, to do those extra cases.”* [Surgery Registrar]

Interviewees emphasised the increased importance of clear, calm and polite communication when under pressure, which can help prevent any further deterioration in working conditions (e.g. interpersonal conflicts). Thought also went into how and when to communicate plans with patients to reduce anticipatory anxiety, disappointment and additional workload for administrative staff communicating changes with patients.

Co-ordinated planning, teamwork and shared decision-making across disciplines, specialties and different sites were critical to be able to respond to increasing pressures. This was mostly achieved through planned multidisciplinary team meetings, as it was not easy to communicate during the day when individuals were in different theatres. It was also important to make allowances for new or covering staff, for example going slowly where needed and allocating roles based on experience rather than hierarchy.

*“And what we did with this meeting was we actually had a cross-divisional meeting, and we pushed that responsibility to every team that is required to prepare the patient, from whichever part of the system they come from. And it’s actually a rehearsal of what’s going to happen in the following two weeks. So everybody is aware. The meeting’s actually attended by Admin, so Admin of different sites, so not just [X], Admin of different sites. It’s attended by theatre nursing staff, it’s attended by anaesthetists, it’s attended by CNSs, it’s attended by dietician and speech-and-language therapists. It’s attended by the Preassessment Clinic. It’s attended by the surgeons and all the fellows.”* [Surgeon]

Leadership strategies were often based around fostering an environment where staff felt they could speak up and pay close attention to the knowledge and skills of your team. Having these features in place helped to protect against problems and errors at times of pressure. Other strategies included stepping back to reassess the situation, focussing on what was within your influence and being more directive and autocratic at times of increased pressure.

### Benefit & downsides to adaptive strategies

Adaptive strategies in the face of pressures and challenges are almost always well-intentioned efforts to meet patient need. However, adaptations can have positive short term effects but also adverse effects in both the short and longer term. Risk-management in the face of everyday pressures often meant having to trade-off safety with other objectives such as efficiency or staff wellbeing.

*“So what do we do? What’s best for the service which is just get on with it [patient operation], what’s best for the patient which is probably defer them for two weeks but it won’t be two weeks, it’ll be two months because everything is full. So it’s patient-focused versus hospital operations side.”* [Anaesthetist and Head of Department]

The three cases studies described above also illustrate the trade-offs between immediate benefit to patients and potential adverse effects in the longer term, though the balance of benefit and risk varies considerably. For example, the case study in Table 2 is mostly beneficial in the sense that it led to a new and potentially more efficient approach to providing sterile services. There is a short-term drawback of releasing staff temporarily but longer-term benefits of improving skills and service efficiency. Whereas, the example in Table 3 has obvious short-term gains of getting a patient through surgery, but adverse effects for recovery staff working outside the scope of their practice, a patient who may be exposed distressing scenes and without access to facilities, and family who are unable to visit their relative. The case study in Table 6 shows how teams will be formed and pulled together at very short notice to manage rapid changes and demand. However, this type of strategy relies on the willingness and availability of staff which, as those interviewed stressed, has its limits. Continual changes to team structures and schedules will quickly become both disruptive and exhausting.

## Discussion

Teams in all areas of healthcare may deploy a range of strategies when under pressure and make adaptations to the way care is delivered. The primary aim is to minimise the risks to patients and maintain a reasonable, if not ideal, quality and safety of care within the available constraints. We recently developed a generic taxonomy of strategies for responding to pressures [8]. This provided the foundation for the present study in which we explored the specific pressures in surgery and the strategies used by clinical leaders and their teams when delivering care under pressure.

The dominant source of pressure reported by the surgical teams in this study is simply that patient demand exceeds the available resources, with a shortage of skilled staff being the most frequently cited problem. When demand exceeds capacity, then working conditions become more difficult which in turn disrupts patient flow which, in a vicious negative feedback loop, makes it more difficult to cope with rising demand. Plans are in place to increase the medical and nursing workforce in the longer term [10] but, in the meantime, clinical teams have to constantly meet the challenge of being unable to deliver either the volume or standard of care that they would ideally like to. The challenge for surgical teams then is how best to adapt care to give the best possible outcomes for patients within the constraints while not placing impossible burdens on staff.

In surgery, many strategies and adaptations are made in advance to anticipate pressures, with a focus on scheduling to improve efficiency coupled with strategies for controlling and prioritising demand. When waiting lists are long, teams constantly have to make difficult decisions about whether or not to cancel surgery. Immediate risks of operating are often easier to assess than the potential risks of delaying the surgery on the patient’s physical and mental wellbeing. Cancelling elective surgery also has to be weighed against the wider impact on the health system and on patients and their families (e.g. deterioration or developing a comorbidity) [11]. For instance, patient liaison teams need to inform and support disappointed patients, theatre managers need to reallocate space, consultant surgeons and anaesthetists would have to reorganise their time and surgical lists, and trainees would have to make up the surgical hours and cases for their training. Leaders under pressure constantly need to make these trade-offs in order to balance competing priorities, such as between patient safety, staff wellbeing and service efficiency. Sometimes responding to pressures in one place inevitably has consequences for another [13].

Teams in surgery also employ a range of other adaptations and adjustments to care alongside cancellations and delays to surgery. The strategy of flexing and adapting the use of equipment and resources is commonly used. Care is often moved to other areas of the hospital. For instance, patients who might normally be in intensive care may be cared for on the wards; patients stay in the recovery suite overnight rather than returning to the ward after an operation; male patients may be allocated to female day surgery wards. Surgical teams also make many adaptations to their usual ways of working. Task shifting for instance is very common, with junior doctors taking on additional responsibilities, or student nurses being trained to take on full clinical roles much earlier than would be usual. Task shifting also extends to patients and families, as early discharge home effectively means shifting clinical tasks and responsibilities, such as monitoring patients and caring for wounds, from nurses on the ward to families in the home [14, 15]. Clinical leaders also employ an additional range of on-the-day adaptive strategies in both wards and theatre, such as a greater emphasis on multi-modal communication to monitor care and detect safety issues and the practice of pausing an operation or care to enable the clinical team to refocus and prioritise. Effective teamwork is vital in surgery and to be effective, all these adaptive strategies need to be co-ordinated between the different professions within the operating team (i.e. surgeons, anaesthetists, scrub nurses), and between the operating team, theatre managers (to allocate theatre space) and ward managers (to allocate bed space) [16, 17].

Adaptations are made by staff in the face of substantial pressures which demonstrates the complexity of decision-making in a stretched system, but at times adaptations lead to major departures in standards outlined in policies. The care provided may then be a long way from the standards of care staff aspire to provide. For instance, some interviewees reported that some practices that formerly would have been considered unthinkable and a patient safety incident, such as caring for a patient overnight in recovery or discharging a patient home from intensive care, are becoming normalised. This is obviously stressful for staff, with the violation of professional norms increasing the risk of ‘moral injury’ and burnout (Wilkinson, 2020). Individual patients may benefit in the sense of having an operation that might otherwise have been cancelled, but at the cost of a very different standard of care, potentially distressing experiences and an increased burden on those caring for the patient at home. There is a critical role for leaders, both executive and clinical, in discussing such compromises openly and supporting teams faced with unenviable decisions [19]. The risk of moral injury will be less if such decisions are seen as a necessary collective decision rather than an individual personal failing [20].

### Strengths and limitations of the study

We purposely selected four different and diverse hospitals to obtain a broad perspective of views. Given multidisciplinary working is a strong feature in surgery, a key strength of this study was that the sample represented a cross-section of different professions involved (albeit not all) and provides a rich understanding of the pressures and strategies used from the different perspectives. We should also note that most participants were relatively senior, as they seemed most able to explicitly acknowledge, describe and initiate adaptations to usual care. Of course, more junior staff also adapt, but they generally have less autonomy to make system level adaptations. There may also be some selection bias in that those who volunteered to be interviewed are potentially more committed to patient safety initiatives and so more aware of risk management strategies. Another limitation is that it is not always possible for people to articulate exactly how they adapt when under pressure and interviewees varied in how well they could describe the strategies they use. It is possible that by using ethnography/observations, additional strategies might be revealed. Strategies will also vary to some degree between different types of surgery which can be explored in future research. A strength of the paper is that the analysis was sense-checked by two clinicians (a surgeon and an anaesthetist), and that the interviews were conducted with two members of the research team present.

### The potential for training to manage under pressure

When pressures are high, a coordinated strategy of balancing resources and demand and managing the workload is likely to be much safer than a fragmented and individualised set of improvisations [7]. The multidisciplinary nature of surgery means it is important to coordinate adaptations across disciplines, aligning objectives and coming to joint decisions. The strategies described here could help clinicians and managers respond to similar pressures, providing a portfolio of strategies that clinical teams could use or develop for their own contexts. The implications of using certain strategies should be considered from multiple perspectives, including service performance, patient risk and impact on staff. Strategies may have very different effects on these various parameters; a strategy may for instance reduce risk for patients but increase burden on staff.

These strategies could be incorporated into training programmes to prepare staff moving into leadership roles or those newly in charge, who are responsible for the functioning of a service and have the authority to guide and support team and system-level adaptations. For example, simulation or scenario-based exercises on prioritisation and managing competing demands could help to reduce stress and uphold safe practices when individuals have to make strategic decisions quickly in pressurised situations [21, 22]. In surgery in particular, training in interprofessional groups or teams may be especially beneficial for sharing expertise and generating discussion of collaborative strategies [23]. Clinical teams can explore their own current approaches, which will vary between individuals, with the aim of achieving a more coordinated approach. Wider, more formal training programmes will require organisations, and indeed regulators, to explicitly acknowledge the difficulties of maintaining standards of care when under pressure and see such training as a necessary form of proactive risk management. Future work will explore what this type of training might look like and how it could be organised, with attention given to efficacy, trade-offs and implications of a menu of strategies.

### The need for further research on adaptive strategies in surgery

All strategies have benefits and risks, and our study design does not allow us to systematically assess the effectiveness or adverse effects of any of the strategies described. For example, staff staying late to complete a list may improve patient safety but will clearly have a negative impact on staff wellbeing. Systems that rely on individuals adapting at maximum capacity every day leave no margin to respond to unusual demands, and there is a limit to the benefit of some strategies especially when used frequently [24]. There may be certain strategies or combinations of strategies that are better than others or have differential trade-offs and impact on safety, staff well-being, patient flow and patient experience [7]. Further research is needed to explore the effectiveness of different strategies and combinations of strategies in surgery. The development of our taxonomy of pressures and strategies and the exploration of such strategies in surgery provides the foundation for describing the portfolio of strategies used in different surgical units and an assessment of their impact and effectiveness.

## Conclusions

Adaptation is a dynamic, unfolding process and strategies are often deployed in combination to respond to the current pressures. Leaders and their teams in surgery deploy a variety of strategies in combination to manage pressure, though these vary considerably between teams and between hospitals. The aim of these strategies is to minimise patient risk and maintain a reasonable volume, quality and safety of care within the available constraints. It is clear however that, while these strategies are necessary and aimed at providing better care overall, they may also involve substantial risks to patients and staff and departures from accepted standards of care. Our aim in this study however is not simply to describe the adaptations but to pave the way for a more open, transparent and coordinated approach to adaptations. We believe that it would be better to develop and implement a portfolio of prepared strategies for managing risk at times when ordinary standards cannot be met and the safety of patients is compromised, than to have multiple individuals adapting in different ways [7]. This study showed that it is possible to identify strategies used by surgical teams which could be shared with clinicians and managers, with potential for training in coordinated adaptations at times of pressure. The recognition of hazards combined with open discussion of risks combined with the development of active, practical risk management strategies is the route to safer healthcare [25].

### Supplementary Information

**Supplementary material 1.**

## Data Availability

No datasets were generated or analysed during the current study.
