# Supplementary material for: Adaptive strategies used by surgical teams under pressure: an interview study among senior healthcare professionals in four major hospitals in the United Kingdom
Source: Patient Saf Surg. 2024 Feb 21;18:8. doi: 10.1186/s13037-024-00390-3 (PMC10880194; doi:10.1186/s13037-024-00390-3)
Supplement: Supplementary file 1 — Supplementary material 1. [file 13037_2024_390_MOESM1_ESM.pdf]

How can people and organisations best respond to pressures on services?

INTERVIEW SCHEDULE

1. Ask about job role, responsibilities and clinical setting
2. Could you please start by describing the types of pressures your team/organisation has experienced recently. If possible, please recall a particular time or situation that illustrates the typical pressures you face.
    a. How did you generally respond to/adapt to the situation?
3. Please tell me about the decisions and choices you have to make when faced with these pressures.
    a. What is your main objective when services are under pressure?
4. Please give some examples of strategies you and your organisation have used when responding to these pressures.
5. Are the changes you've described planned in advance? Are they ad hoc adjustments? Or are they a mixture of both planned and ad hoc adjustments?
6.
    a. How are these changes communicated to patients and staff?
    b. Do you introduce any specific additional support for staff at times of pressure?
7. What are the benefits and downsides to the changes to services you have described? Please share with me how, if at all, the pressures you have described affected the care/service you provided to patients, staff, organisation and wider health system.
8.
    a. How have you developed the strategies you use to manage when pressures are high?
    b. What have you learned that others could adopt to ensure that pressures do not impact on patient or staff safety?
    c. What are the top three things you would advise others to do that enables them to maintain safety on a shift when pressures are high?

These topics are a guide to stimulate naturalistic conversation that will be probed further depending on the responses of each individual participant.
